# Nonlinear variation of discharge coefficient and energy dissipation optimization for bottom outlet of EG reservoir: an integral hydraulic model study

**DOI:** 10.1038/s41598-026-49463-6

**Published:** 2026-04-22

**Authors:** Ying Li, Jiwei Zhao, Yongshuai Yan, Shihao Shen, Xiaolei Zhang, Quansheng Luo

**Affiliations:** 1 North China University of Water Resources and Electric Power, Zhengzhou, 450046 China; 2 Yellow River Conservancy Technical University, Kaifeng, 475004 China; 3 Zhengzhou University, Zhengzhou, 450001 China; 4 Henan Provincial Engineering Research Centre for Giant Water Network Disaster Prevention, Kaifeng, 475003 China; 5 School of Hydraulic Engineering, Yellow River Conservancy Technical University, Kaifeng, 475004 China

**Keywords:** Hydraulic model test, Discharge coefficient, Nonlinear characteristics, Energy dissipation optimization, Bottom outlet, Stilling basin, Energy science and technology, Engineering, Hydrology

## Abstract

Precise quantification of discharge capacity is essential for flood routing and reservoir safety. However, conventional design practices commonly assume a constant discharge coefficient (*µ*), neglecting the nonlinear variations that emerge during partial gate operations. This simplification introduces systematic errors in hydrological calculations and compromises downstream flow stability. This study employs a 1:45 geometrically undistorted hydraulic model of the EG Reservoir to investigate the nonlinear evolution of the bottom outlet discharge coefficient and to optimize the associated energy dissipation system. Experimental results reveal a pronounced “*U*-shaped” variation in *µ* relative to gate opening (*G*). While small openings (*G* < 2.0 m) maintain high discharge efficiency (*µ =* 0.77–0.82), a hydraulically “sensitive zone” emerges at intermediate openings (2.0 m < *G*<3.0 m). In this zone, intensified vertical jet contraction drives *µ* down to a minimum of 0.67, approximately 21% lower than standard design code recommendations. Accordingly, a cubic polynomial correction model (R^2^ > 0.99) was derived for real-time discharge prediction. This study provides a systematic quantification of the *U*-shaped nonlinear evolution mechanism of the discharge coefficient with gate opening for the radial-gated bottom outlet of the EG Reservoir and quantifies the discharge deviation amplitude (up to approximately 21%) in the hydraulic sensitive zone (*G* = 2.0–3.0 m). To mitigate the hydraulic instability exacerbated by these discharge fluctuations, the stilling basin geometry was optimized by deepening the apron (from 5.0 m to 7.4 m) and raising the end sill. This geometric optimization transformed the flow regime from an unstable swept-out jump to a stable submerged jump, increasing energy dissipation efficiency from 46.3% to 64.5% under design flood conditions. These findings establish a quantitative framework for refined gate operation protocols and resilient energy dissipation design, with the correction model applicable for gate openings *G* = 0.96–4.80 m (R² > 0.99) under the tested geometric conditions, and demonstrated downstream scour reduction of 35–40%.

## Introduction

Flood discharge safety constitutes a core technical challenge in hydraulic engineering, depending fundamentally on the precise calculation of discharge capacity. In the design of flood release structures, the discharge coefficient (*µ*) is the pivotal parameter determining calculation accuracy, directly influencing reservoir routing and downstream flood control decisions^1–3^. For reservoirs equipped with bottom outlets, these structures serve multiple functions including reservoir emptying, sediment flushing, and participation in flood discharge operations. Consequently, accurate prediction of their discharge capacity is of critical importance for reservoir operation and management. However, the hydraulic behavior of bottom outlets under partial gate openings is inherently complex. Traditional design approaches apply a constant coefficient, failing to account for dynamic changes in flow boundaries and contraction effects as the gate position varies. This simplification constitutes a major bottleneck in refined flood scheduling.

Hydraulic model testing remains an essential methodology for investigating the hydraulic characteristics of flood discharge structures. Ensuring that laboratory-scale results faithfully represent prototype behavior requires careful attention to scale effects and similarity criteria. Regarding methodological foundations, Heller^4^ systematically reviewed scale effects in physical hydraulic models, establishing guidelines for minimizing distortions in Froude-scaled experiments. Chanson^5^ presented a rigorous treatment of open channel flow hydraulics, establishing the theoretical framework for energy dissipation analysis and hydraulic jump characterization that underpins modern physical model design. Wang and Zhang^6^ conducted experimental investigations on control schemes for riverbed erosion at stilling pool ends, demonstrating how tailwater channel protection measures can be optimized through systematic model testing. In the domain of energy dissipation, scholars have examined the vibration characteristics of discharge structures induced by hydraulic jumps during high-dam flood discharge, revealing the dynamic coupling between turbulent flow pulsation and structural response^7,8^. Wei et al.^9^ performed experimental studies on energy dissipation and tailwater wave characteristics in two-stage stilling basins with supercritical inflow at low Froude numbers, providing important reference data for stilling basin design optimization. Concurrently, numerical simulation methods have provided powerful supplements to physical model testing: Zhong et al.^10^ and Aydin et al.^11^ analyzed flow characteristics in flood discharge stilling basins with step-down floors, validating the applicability of numerical approaches for capturing complex recirculation patterns. Collectively, these advances have broadened the analytical toolkit for investigating the discharge characteristics of flood release structures.

Nonlinear discharge coefficient variation for bottom outlets and gated structures has attracted considerable attention in the hydraulic engineering community. Swamee^12^ derived analytical discharge equations for sluice gates that captured the nonlinear relationship between gate opening ratio and discharge coefficient, establishing a theoretical foundation for understanding flow contraction effects under varying gate positions. Subsequent computational and experimental studies have further elucidated how gate geometry influences discharge nonlinearity. For radial (tainter) gates, researchers have demonstrated that the curved gate lip introduces additional flow deflection compared to planar gates, producing more pronounced coefficient variations at intermediate openings^13–15^. In China, experimental investigations on major reservoir bottom outlets have documented similar nonlinear coefficient behaviors under partial gate operations, although systematic quantification of the underlying mechanisms remains limited^16,17^. Numerical simulation approaches using Reynolds-Averaged Navier–Stokes (RANS) models and Large Eddy Simulation (LES) have been applied to predict discharge coefficient variations, with reasonable agreement against experimental data for specific gate configurations^10,11,14,18,19^.

Despite considerable progress in hydraulic model testing, significant gaps remain in understanding the controlled discharge characteristics of bottom outlets, primarily in two aspects: First, the variation of bottom outlet discharge coefficients with gate opening has not been systematically elucidated. Current engineering practice commonly employs constant discharge coefficients to estimate bottom outlet discharge capacity, a simplification originating from early experimental limitations and engineering safety margin considerations. However, this approach neglects the significant changes in flow boundary conditions during partial gate opening: as gate opening varies, the cross-sectional geometry, degree of flow contraction, and turbulence characteristics undergo corresponding changes, causing the discharge coefficient to exhibit nonlinear variation. Miao et al.^20^ investigated the discharge capacity of pressurized tunnels under different gate openings through theoretical analysis and model testing, establishing a preliminary quantitative relationship between opening and discharge, providing a useful benchmark for understanding variable-opening discharge characteristics of bottom outlets. Nevertheless, that study focused primarily on the macroscopic trend of discharge variation with opening, without adequately characterizing the evolution of the discharge coefficient itself. Specifically, it failed to reveal the “*U*-shaped” nonlinear evolution mechanism of the discharge coefficient (i.e., the characteristic of initial decrease followed by increase), nor did it identify the “sensitive zone” phenomenon of rapid coefficient decline in the intermediate opening range (e.g., *G* = 2.0–3.0 m). Although the aforementioned previous studies^13–19^ established important macroscopic discharge–opening relationships or focused on specific gate types in isolation, none have quantified the specific *U*-shaped evolution mechanism with defined sensitive zone boundaries for radial-gated bottom outlets, nor have they developed high-precision correction models suitable for direct engineering application.

Second, the coupling between nonlinear discharge characteristics of bottom outlets and energy dissipation performance has not received adequate attention. Changes in bottom outlet discharge conditions affect not only discharge calculation accuracy but also produce cascading effects on the performance of downstream energy dissipation structures. When bottom outlets operate at intermediate openings where the discharge coefficient varies rapidly, the resulting fluctuations in outflow discharge may exacerbate hydraulic jump instability within the stilling basin, leading to reduced energy dissipation efficiency and intensified scour at the pool floor^21^. However, existing research on energy dissipation design has largely been based on fixed discharge conditions, treating the bottom outlet as a stable inflow boundary without systematic examination of the intrinsic connection between discharge coefficient variations and energy dissipation performance.

To address these gaps, this study conducts a systematic experimental investigation using a 1:45 geometrically undistorted hydraulic model of the EG Reservoir. The research treats bottom outlet discharge characteristics and energy dissipation optimization as interrelated aspects of an integrated problem. The present work advances beyond existing research in three respects: (i) it reveals the specific *U*-shaped evolution mechanism with quantified sensitive zone boundaries; (ii) it develops a high-precision polynomial correction model for direct engineering application; and (iii) it couples the discharge coefficient variation with downstream energy dissipation optimization—an integration not addressed in prior literature. The objectives are to: (1) quantify the nonlinear “*U*-shaped” variation of *µ* and identify the “sensitive zone”; (2) derive a high-precision correction model for engineering applications; and (3) propose a geometric optimization scheme (“deepened pool floor + raised end sill”) to mitigate hydraulic instability caused by discharge fluctuations.

## Materials and methods

### Project overview

**Fig. 1:**
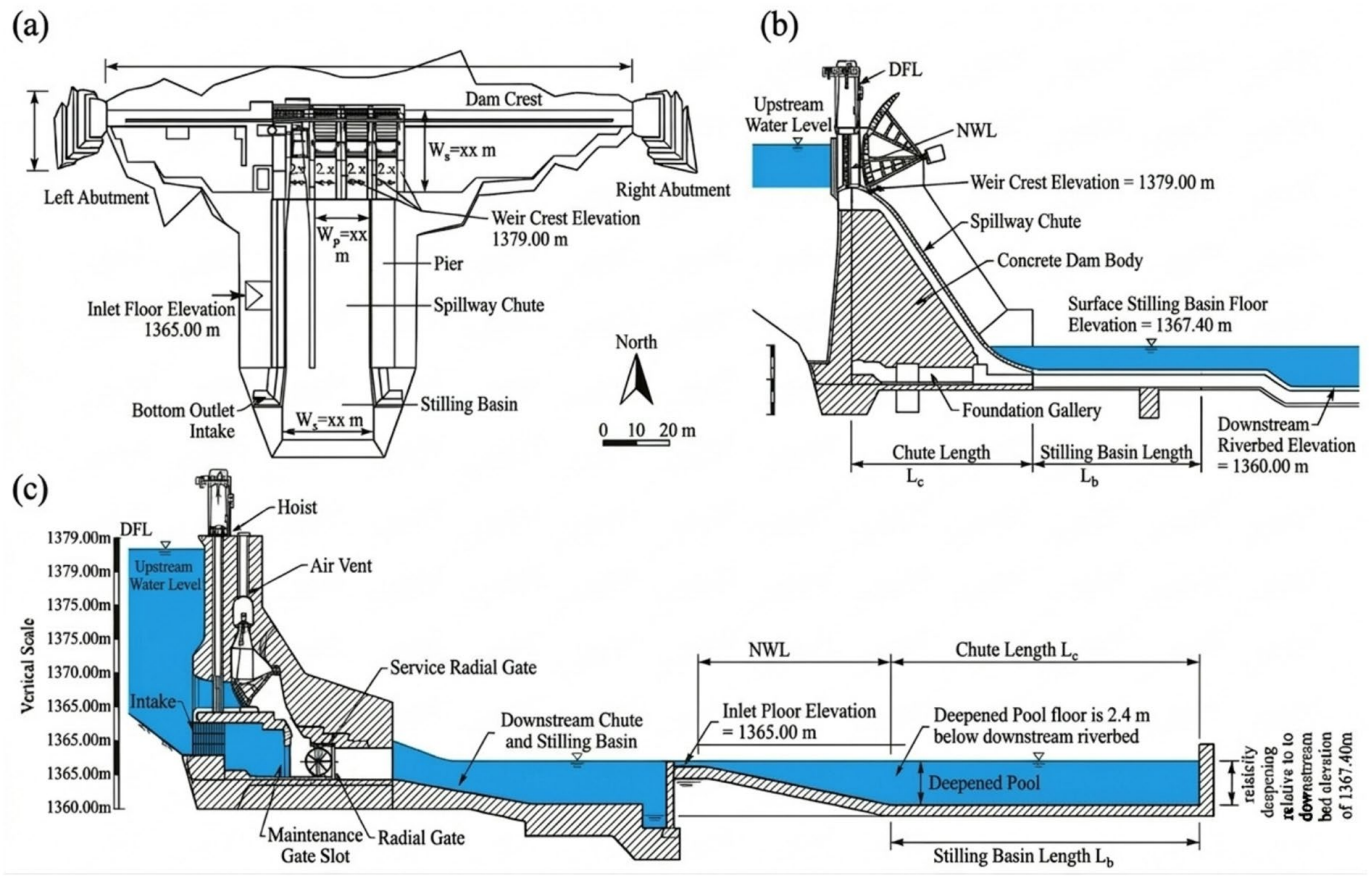
(**a**) General layout of the project; (**b**) Elevation drawing of the surface outlet dam section; (**c**) Elevation of the bottom outlet dam section.

The EG Reservoir is situated in the upper reaches of the Yellow River basin in a mountainous region of western Henan Province, China. The catchment area is approximately 126 km², with a mean annual runoff of 32 × 10⁶m³. The flood season extends from June to September, during which the reservoir experiences moderate sediment loads typical of tributaries in this region. EG Reservoir is a small (Type 1) mountain impoundment reservoir with a total storage capacity of 9.05 × 10⁶m³, classified as Grade IV project. The dam comprises non-overflow dam sections, surface outlet spillway sections, bottom outlet dam sections, and water intake dam sections, totaling four dam segments. The surface outlet spillway section is positioned in the main riverbed, incorporating two surface outlets employing hydraulic jump energy dissipation. The bottom outlet section is located immediately to the right of the surface outlets, with one bottom outlet designed for flood discharge and sediment flushing. The inlet floor elevation is 1378.00 m, with outlet dimensions of 4.5 m (width) × 4.8 m (height), controlled by a radial gate and utilizing hydraulic jump energy dissipation. The surface and bottom outlet stilling basins are separated by a dividing wall (Fig. 1).

The bottom outlet is equipped with a radial (tainter) gate with a radius of 6.0 m and a trunnion pin elevation of 1382.80 m. The gate lip features a sharp-edged profile with a curvature radius of 15 mm. At the design maximum opening of *G* = 4.80 m, the angle between the gate lip tangent and the tunnel floor is approximately 53°; at intermediate openings (*G* = 2.0–3.0 m), this angle ranges from 28° to 38°, corresponding to the range where vertical flow contraction is most pronounced. These geometric parameters are essential for understanding the physical mechanism of the *U*-shaped discharge coefficient variation described in Sect. 3.

### Model design, construction, and similarity criteria

A rigid-bed, geometrically undistorted physical model was constructed from steel, acrylic glass, concrete, and timber. The spillway sections and gate piers were fabricated from acrylic glass with geometric precision controlled to within 0.2 mm, while upstream and downstream river channel topography (fixed bed) was formed using the stake-point layout method. The model extent was determined to ensure flow patterns in the test section maintain similarity with the prototype while avoiding boundary condition interference with reservoir flow. Considering factors including maximum flood water level with freeboard, discharge measurement facility arrangement, and downstream scour depth, the model simulation extent was established as: upstream river channel extending approximately 240 m (prototype length, same below) from the dam site to elevation 1416 m; cross-valley width of 320 m upstream of the dam to ensure sufficiently broad approach flow boundaries at the flood discharge structure inlets; downstream river channel simulated to approximately 350 m beyond the stilling basin tailwater channel, to topographic elevation 1380 m. The model encompasses upstream and downstream river channels, surface outlet dam sections, bottom outlet dam sections, and portions of non-overflow dam sections, comprehensively representing the flow characteristics of the flood discharge structures.

A rigid integral model is adopted because the deformation of the concrete gravity dam under flood discharge conditions is negligible. The maximum dam height is approximately 46 m, and structural analysis confirms that hydraulic loading-induced deformations are on the order of millimeters at prototype scale, which at 1:45 scale translates to sub-micrometer displacements, well below the geometric precision of the model (0.2 mm). Fluid–structure interaction effects are therefore excluded from this investigation. This rigid model assumption is standard practice for concrete gravity dams of this scale in accordance with Chinese design codes and ASCE guidelines for physical hydraulic modeling.

Following hydraulic model similarity theory, model design must satisfy gravitational similarity criteria, i.e., Froude number equality, ensuring identical ratios of inertial to gravitational forces between model and prototype flows. Considering engineering characteristic dimensions and laboratory facility constraints, an undistorted scale approach was adopted with a geometric scale ratio of *λ*_*L*_ = 45. The corresponding scale relationships are presented in Table 1.

**Table 1:** Model scale conversion relationships.

Physical quantity	Scale formula	Scale value
Geometric scale	*λ*_*L*_	45
Velocity scale	*λ*_*v*_ = *λ*_*L*_^0.5^	6.7
Discharge scale	*λ*_*Q*_ = *λ*_*L*_^2.5^	13,583
Time scale	*λ*_*t*_ = *λ*_*L*_^0.5^	6.7
Roughness scale	*λ*_*n*_ = *λ*_*L*_^(1/6)^	1.88

### Model validation

This model primarily investigates reservoir flood discharge flow patterns, discharge capacity, and energy dissipation problems, with flow motion dominated by gravitational effects, thus employing Froude criteria for model design. To ensure reliability of model test results and avoid significant distortion from excessive scale reduction, the following key hydraulic parameters were verified:

(1) Viscous force influence verification: According to similarity theory, when model flow enters the quadratic resistance regime (fully turbulent), viscous force effects become negligible. Under minimum test head conditions, the calculated Reynolds number for the surface outlet spillway is *Re* = 4.8 × 10⁴, far exceeding the critical Reynolds number (generally requiring *Re* > 4000), indicating that model flow remains in the turbulent state throughout, satisfying resistance similarity requirements.

(2) Viscous force influence verification for the pressurized flow section: Given that the bottom outlet involves a significant pressurized flow section, an additional Reynolds number verification was performed specifically for the pressurized tunnel. Under minimum test head conditions, the calculated model Reynolds number for the pressurized tunnel section is *Re* = 3.2 × 10⁴, which substantially exceeds the critical value (Re > 4000 for fully turbulent pipe flow). This confirms that the model flow in the pressurized section operates in the fully turbulent regime, satisfying resistance similarity requirements and ensuring that viscous scale effects do not significantly distort the measured discharge coefficients.

(3) Surface tension influence verification: To eliminate surface tension effects on overflow capacity and water surface profiles, model overflow depth must satisfy certain restrictions. In this study, the minimum overflow depth at the surface outlet weir crest exceeds 2.0 cm, with corresponding Weber number *We* = 130, satisfying the condition for neglecting surface tension (*We* > 100) and ensuring authenticity of water surface profile simulation.

(4) Roughness similarity control: The prototype spillway surface is concrete with a roughness coefficient of approximately 0.015. Calculations indicate the corresponding model components require a roughness of 0.008. High-precision acrylic glass was used for spillway sections and gate piers, with a roughness coefficient of approximately 0.008, essentially matching the calculated value and satisfying flow resistance similarity requirements.

### Instrumentation and measurement

Water levels were measured using fixed point gauges with precision controlled to ± 0.2 mm; each measurement was repeated 2–3 times with stable or averaged values recorded at ± 0.3 mm reading precision. Water surface profiles were measured using a movable point gauge (with level) at ± 0.3 mm precision. Flow velocities were measured using direct-reading photoelectric propeller current meters with ± 2% precision. Time-averaged pressures were measured using piezometer tubes with ± 0.5 mm precision. All measurement instruments were calibrated and certified within their validity periods.

**Fig. 2:**
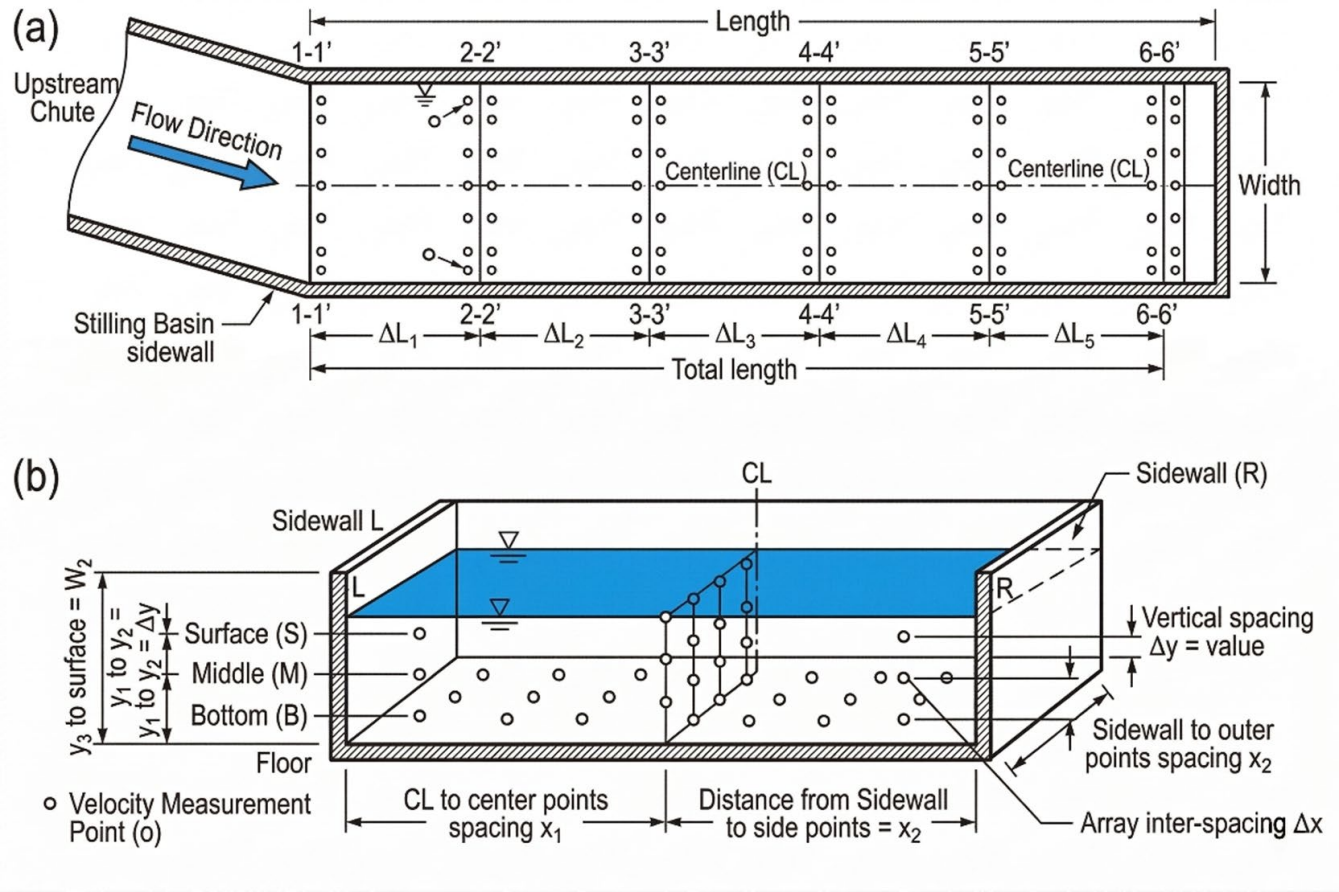
Layout and detailed spatial distribution of velocity measurement points in stilling basin spatial layout of velocity measurement cross-sections. (**a**) spatial layout of velocity measurement cross-sections. (**b**) detailed cross-section (e.g., Sects. 3–3) looking downstream.

Flow velocities were measured using LS300-A direct-reading photoelectric propeller current meters (range: 0.01–4.00 m/s, accuracy ± 2%). Each measurement point was sampled for a duration of 60 s to ensure adequate temporal averaging and minimize random error in velocity readings. Each measurement point was sampled three times, with the final value taken as the arithmetic mean. Velocity measurement point layout followed principles of representativeness, systematicity, and uniformity. For the original design stilling basin, five cross-sections were arranged from upstream of the gate piers to the end of the spillway for the surface outlet, with the latter four sections uniformly spaced. Four cross-sections were uniformly distributed within the stilling basin at intervals of 8.0 m (prototype)/0.178 m (model); for the first three sections, due to greater water depths, measurement points were arranged at surface, middle, and bottom positions in the vertical direction, with vertical spacing between measurement points of 1.5 m (prototype)/33.3 mm (model). Horizontal positions were set at the basin centerline and at 1/4 width from each sidewall (Fig. 2).

### Test conditions

Tests were based on actual engineering design flood and check flood conditions, with multiple typical operating scenarios established to simulate flood discharge processes under different gate opening combinations. Key test conditions included: *P* = 5% flood with bottom outlet partial opening (discharge 271 m³/s, reservoir level 1412 m); *P* = 2% design flood with bottom outlet fully open and surface outlet partially open (total discharge 617 m³/s, reservoir level 1412 m); and *P* = 0.2% check flood with both surface and bottom outlets fully open (total discharge 1548 m³/s, reservoir level 1412.83 m). The main experimental conditions are listed in Table 2.

In addition to the above flood conditions, supplementary tests were conducted at small gate openings (0.96 m) under the normal water level to cover the full practical operating range of the bottom outlet. These additional data points extend the lower bound of the correction model and confirm that the discharge coefficient at very small openings follows the high-efficiency trend described in Sect. 3. The design tail water levels for the three flood frequency conditions were determined from the stage–discharge rating curve of the downstream river channel, which is derived from hydrological survey data and flood frequency analysis of the basin. Specifically, the tail water levels correspond to the computed downstream water surface elevations associated with the respective flood discharges at the *P* = 5%, *P* = 2%, and *P* = 0.2% frequencies.

The model test was carried out under clear water conditions. The influence of sediment transport on the discharge coefficient and energy dissipation performance will be investigated in future work through sediment–water coupling experiments.

**Table 2:** Key test conditions for combined operation of surface and bottom outlets.

Flood frequency (*P*)	Total discharge(m^3^/s)	Surface outlet discharge(m^3^/s)	Bottom outlet discharge(m^3^/s)	Upstream water level(m)	Operation mode
*P* = 5%	271	/	271	1412	Bottom outlet: partial opening
*P* = 2% (design Flood)	617	151	466	1412	Surface outlet: partial opening
*P* = 0.2% (check flood)	1548	175	473	1412.83	Surface and bottom outlets: fully open

## Results

### Surface outlet discharge capacity

The spillway surface outlets serve as the primary flood discharge structures, carrying the principal discharge load during high-flow events. According to SL319-2018 “Design Code for Concrete Gravity Dams”^22^, the discharge calculation formula for open overflow weirs is:

1 $$\:\mathrm{Q=}\rm{c}{\cdot}{\epsilon}{\cdot}{\sigma}{s}{\cdot}\mathrm{m}{\cdot}\rm{n}{\cdot}\rm{b}{\cdot}\sqrt{2g}{\cdot}{H}^{1.5}$$

where *c* is the upstream weir face influence coefficient; *ε* is the lateral contraction coefficient; $\:\rm{\sigma}\mathrm{s}$ is the submergence coefficient; m is the free discharge coefficient; *n* is the number of gate openings; *b* is the net width per opening (m); *H* is the head upstream of the weir including approach velocity (m); and g is gravitational acceleration (m/s²).

**Fig. 3:**
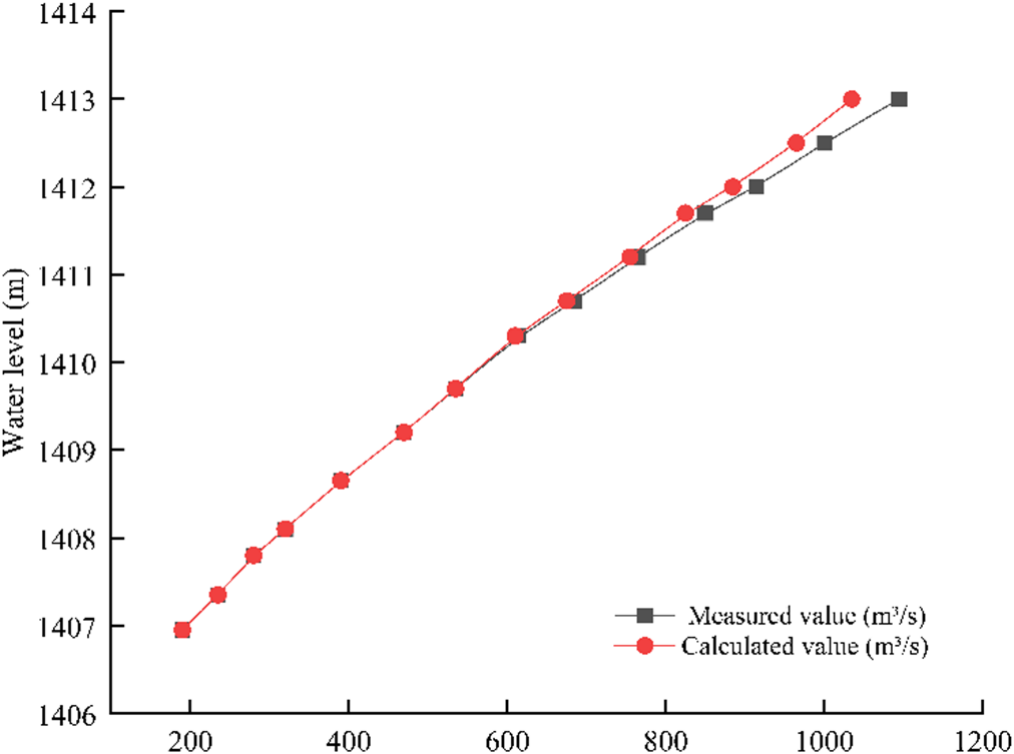
Relationship curve between reservoir water level *Z* (m) and discharge *Q* (m³/s) for the surface outlet under independent free discharge conditions.

The relationship curve between reservoir water level and discharge for independent surface outlet operation (Fig. 3) shows that reservoir water level increases nonlinearly with discharge, indicating ample surface outlet discharge capacity with gradual regulation characteristics. Comparative analysis between calculated curves and model test measurements shows: mean relative error of 1.78%, maximum error of 4.87%, and minimum error of only 0.04%. The Root Mean Square Error (RMSE) is 5.8 m³/s and the Nash–Sutcliffe Efficiency Coefficient (NSE) = 0.987, confirming excellent model–prototype agreement. These errors fall within acceptable ranges, demonstrating good agreement between model test results and design calculations, thereby validating the reliability of the design scheme.

### Bottom outlet full-opening discharge capacity

According to SL319-2018^22^, for deep inlet tunnel outlets with gates partially open under free discharge conditions, discharge capacity is calculated as:

2 $$\:\mathrm{Q}\mathrm{=}{\mu}{\mathrm{A}}_{\mathrm{k}}\sqrt{\mathrm{2}\mathrm{g}{\mathrm{H}}_{\mathrm{w}}}$$

where *µ* is the orifice discharge coefficient (0.83–0.93 for short pressurized deep outlets, with *µ* = 0.86 adopted for this project); *Q* is discharge (m³/s); *A*_*k*_ is the outlet cross-sectional area (m²); and *H*_*w*_ is the head difference between upstream and downstream water levels (m).

**Fig. 4:**
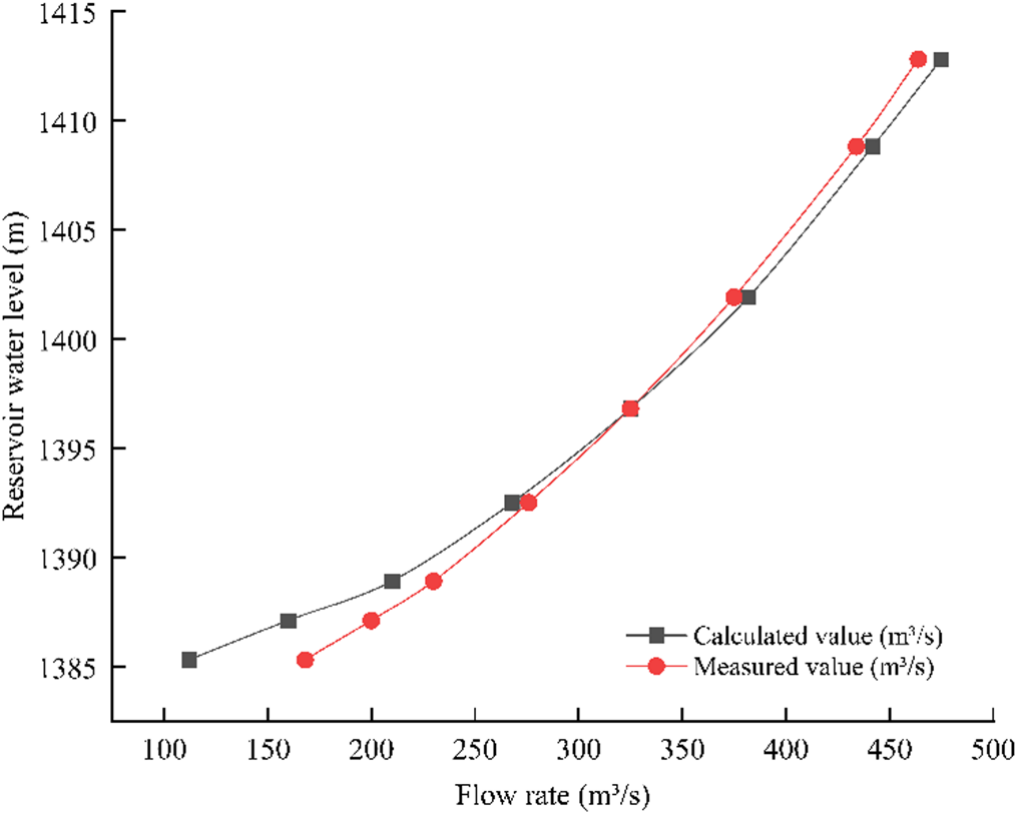
Relationship curve between reservoir water level Z (m) and discharge Q (m³/s) for the bottom outlet under independent fully open operation.

Test results (Fig. 4) indicate that the bottom outlet achieves a maximum discharge capacity of approximately 480 m³/s under fully open gate conditions, corresponding to an upstream water level of approximately 1413 m. This demonstrates substantial flood discharge capability, enabling rapid reservoir drawdown during high-water events to ensure dam safety. Comparison with code-calculated values shows the measured discharge coefficient for the fully open bottom outlet ranges from 0.82 to 0.86, essentially falling within the code-recommended range of 0.83–0.93, validating the credibility and accuracy of the model tests.

### Bottom outlet controlled discharge characteristics

#### Discharge coefficient calculation methodology

Bottom outlets serve multiple functions including flood discharge, sediment flushing, water level regulation, and ecological base flow release. Under certain circumstances (e.g., mitigating downstream scour, structural damage, or coordinated scheduling requirements), adjustment of the bottom outlet gate opening is necessary to balance safe flood discharge with downstream protection.

According to NB-T10391-2020 “Design Code for Hydraulic Tunnels”^23^, for deep inlet tunnel outlets with gates partially open under free discharge conditions, discharge capacity can be calculated as:

3 $$\:\mathrm{Q}\mathrm{=}{\mu}{\cdot\mathrm{A}}_{\mathrm{c}}\cdot\sqrt{\mathrm{2}\mathrm{g}\cdot{\mathrm{(}\mathrm{H}}_{\mathrm{0}}\mathrm{-}{\epsilon}\mathrm{G}\mathrm{)}}$$

where *µ* is the orifice discharge coefficient; *A*_*c*_ is the control section area at the outlet (m²); *ε* is the vertical contraction coefficient; *G* is the gate opening (m). and *H*_*0*_ is the acting head (m). The discharge coefficient can be back-calculated as:

4 $$\:{\mu}\mathrm{=}\frac{\mathrm{Q}}{{\mathrm{A}}_{\mathrm{c}}\cdot\:\sqrt{\mathrm{2}\mathrm{g}\mathrm{(}{\mathrm{H}}_{\mathrm{0}}\mathrm{-}\rm{\epsilon{G}}\mathrm{)}}}$$

**Fig. 5:**
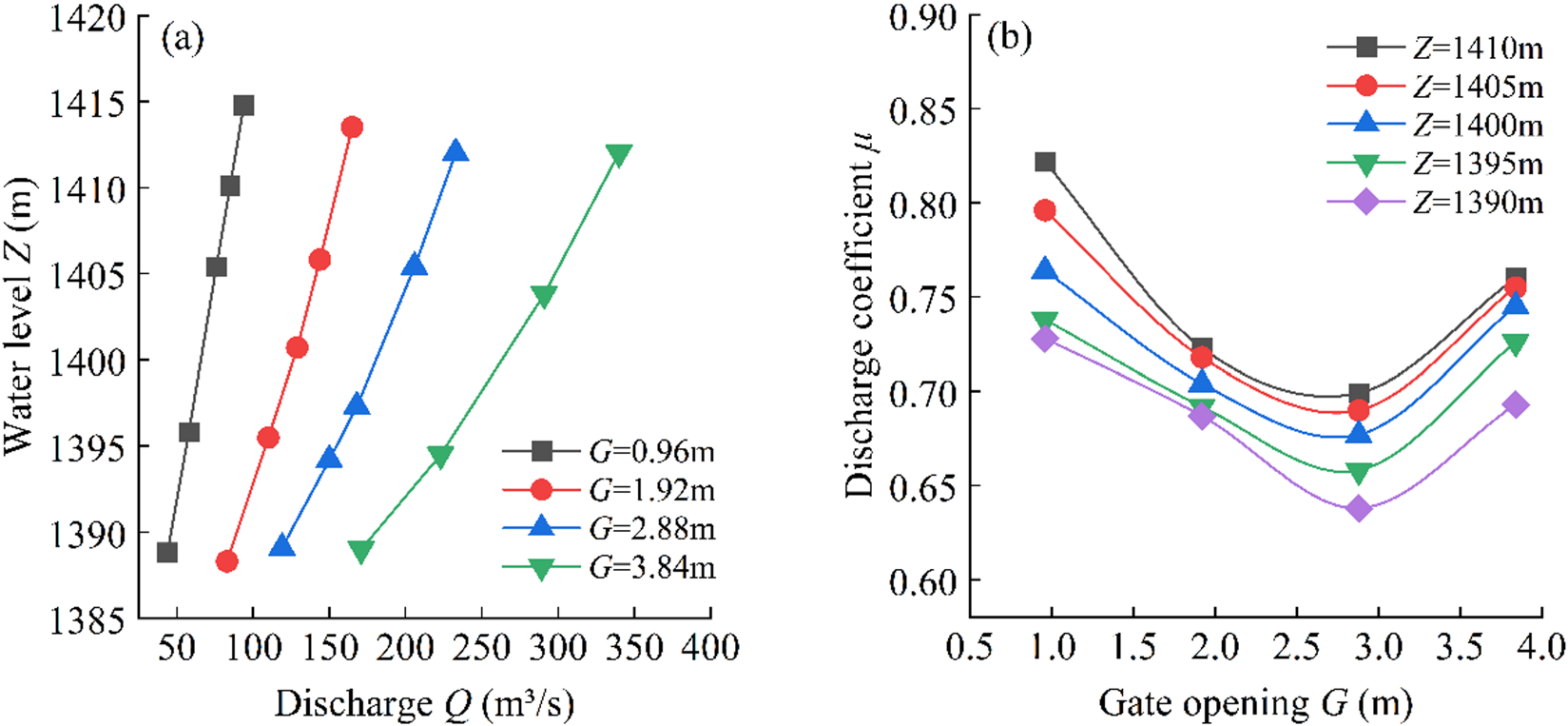
(**a**) Fitted curves of discharge *Q* (m³/s) versus reservoir water level Z (m) at gate openings G = 0.96 m, 1.92 m, 2.88 m, and 3.84 m; horizontal dashed lines indicate reference water levels Z = 1390, 1395, 1400, 1405, and 1410 m used for interpolation. (**b**) Discharge coefficient *µ* versus gate opening G (m) at five reservoir water levels, illustrating the *U*-shaped nonlinear variation pattern.

Through a series of model tests, the water level-discharge relationships for the bottom outlet at gate openings of *G =* 0.96 m, 1.92 m, 2.88 m, and 3.84 m were determined (Fig. 5a).

#### Nonlinear variation pattern of discharge coefficient

Results demonstrate that for bottom outlets with radial gates, the discharge coefficient *µ* and gate opening *G* do not maintain a constant relationship but instead exhibit significant nonlinear variation. At small gate openings (*G* = 0.96 m), the discharge coefficient increases slightly with rising upstream water level, ranging approximately 0.727–0.822. At intermediate gate openings (*G* = 2.88 m), the discharge coefficient reaches minimum values across all water levels, ranging approximately 0.638–0.697. At large gate openings (*G* = 3.84 m), the discharge coefficient shows recovery relative to intermediate openings, ranging approximately 0.691–0.760. Collectively, the data across all openings (Fig. 5b) reveal that the bottom outlet discharge coefficient exhibits a “*U*-shaped” variation trend of initial decrease followed by increase with increasing gate opening.

Under partial gate opening conditions, the measured discharge coefficients deviate significantly from the constant code-recommended value of approximately 0.86. At gate opening *G* = 2.88 m, the minimum discharge coefficient is approximately 0.67, representing approximately 21% lower actual discharge compared to calculations using the constant code value *µ* = 0.85. The comparisons with existing literature are presented in the Discussion section (Sect. 4).

#### Discharge coefficient correction model

**Fig. 6:**
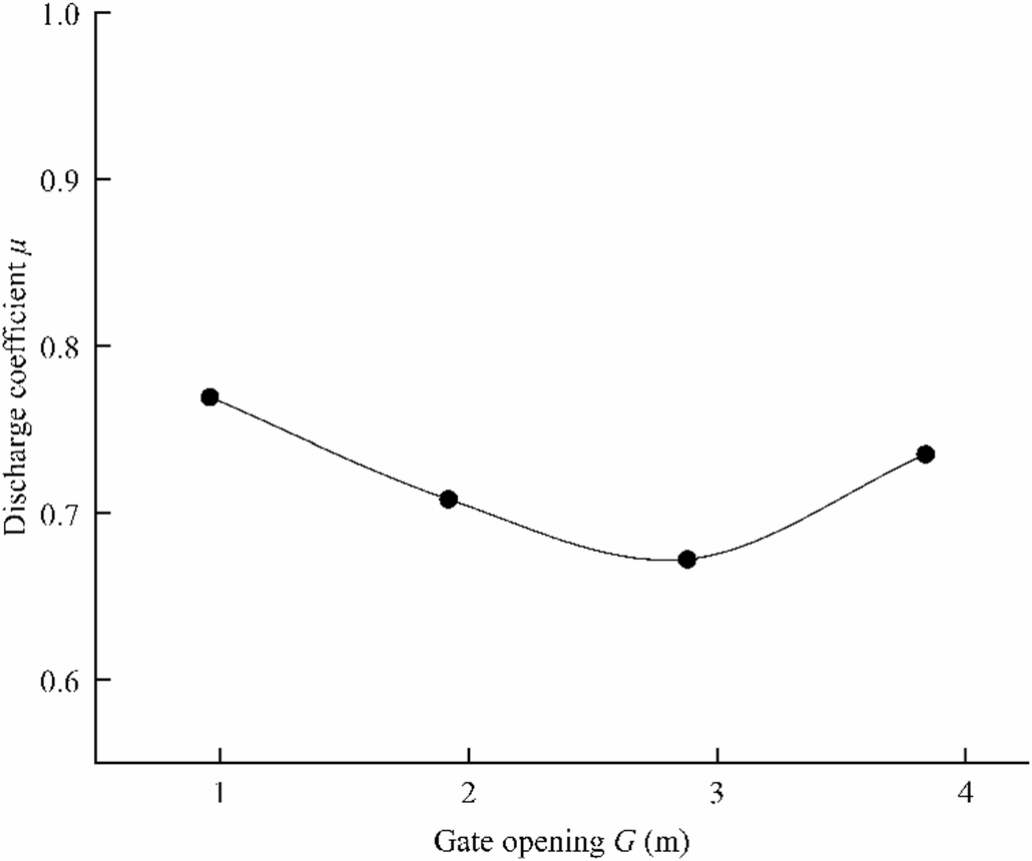
Fitted curve for bottom outlet controlled discharge coefficient *µ* versus gate opening *G* (m). Data points from five reservoir water levels are shown with distinct markers; the solid curve represents the cubic polynomial fit (R² > 0.99).

To establish a quantitative relationship between discharge coefficient and gate opening for engineering applications, test data across different water level conditions were compiled and fitted globally using a cubic polynomial (Fig. 6), yielding the following empirical formula:

5 $$\:\mu\:=0.7814+0.03875G-0.06706{G}^{2}+0.01405{G}^{3}$$

This formula is applicable to deep short-tube bottom outlets with radial gate control, for gate openings *G* in the range 0.48–4.80 m (fully open) and upstream reservoir water levels 1390–1410 m. Extrapolation beyond these ranges requires caution and re-calibration through additional testing. The coefficient of determination R²>0.99 indicates the formula provides excellent fit to test data. The Root Mean Square Error (RMSE) is 0.012 and the Nash–Sutcliffe Efficiency Coefficient (NSE) = 0.991, further substantiating the predictive accuracy of the proposed formula.

Differentiating with respect to *G* and setting the derivative equal to zero yields: Giving solutions *G*₁=2.88 m and *G*₂=0.32 m. Since *G*₂=0.32 m falls outside the valid opening range (below the minimum test opening of 0.96 m), attention focuses primarily on *G* = 2.88 m. Calculation results indicate that when gate opening ranges from approximately 1.0 m to 2.88 m, increasing gate opening strengthens the gate’s obstruction of flow, reducing the discharge coefficient; at *G* = 2.88 m, the minimum value of 0.67 is reached. As opening continues to increase, flow control conditions gradually transition from orifice flow to weir flow, the controlling effect of the gate lip diminishes, and *µ* shows recovery. A comparison between the measured and standard discharge coefficients for different gate opening ranges is presented in Table 3.

**Table 3:** Comparison of measured discharge coefficients with code-recommended values for different gate opening ranges.

Gate opening range	Measured µ	Recommended µ	Relative deviation
Small opening(*G* < 2.0 m)	0.73—0.82	0.85	−4%— −14%
Medium opening(*G* = 2.0 ~ 3.0 m)	0.64—0.70	0.85	−18%— −25%
Large opening(*G* > 3.0 m)	0.69—0.76	0.85	−11%—−19%
Fully open(*G* = 4.8 m)	0.82—0.86	0.83 ~ 0.93	Good agreement

#### Hydraulic sensitive zone identification and control strategies

The physical mechanism underlying the “*U*-shaped” nonlinear variation of discharge coefficient *µ* with gate opening *G* is primarily governed by dynamic interactions between gate lip geometry and flow contraction effects, which can be explained in three stages:

(1) “High-efficiency flow” mechanism in the small opening range (*G* < 2.0 m): In this range, the gate opening height is relatively small, the angle between the radial gate lip tangent and the floor is low, and the flow streamline curvature downstream of the gate is gradual. Vertical contraction of the downstream jet is modest, lateral contraction has not fully developed, and flow boundary obstruction effects are weak, resulting in relatively high discharge coefficients (*µ* approximately 0.77 or above).

(2) Strong contraction mechanism in the intermediate opening range (2.0 m < *G* < 3.0 m): As the gate opens further, the radial gate lip tangent angle increases, forcing abrupt downward deflection of the flow streamlines. Vertical contraction intensifies significantly, the effective flow cross-sectional area decreases sharply, and the discharge coefficient drops to its minimum (*µ* ≈ 0.67). This range constitutes the zone most sensitive to gate opening, termed the hydraulic “sensitive zone”, where the gate lip exerts maximum contraction on the flow.

(3) “Recovery” mechanism in the large opening range (*G* > 3.0 m): As the gate approaches the fully open position, the flow regime transitions from typical short-tube orifice flow toward weir flow. The throttling effect of the gate lip diminishes, the guiding effects of the sidewalls and floor become dominant, jet contraction decreases, and the discharge coefficient recovers with increasing opening.

Based on this analysis, the following engineering control strategies are recommended: if conventional constant coefficients (*µ* = 0.85) are used for flood discharge scheduling, actual discharge within the sensitive zone 2.0 m < *G*<3.0 m will be approximately 20% lower than commanded discharge, potentially causing significant scheduling deviations. Accordingly, two measures are recommended: (1) embedding the discharge coefficient correction formula into dam scheduling SCADA systems to correct commanded discharge in real time based on gate opening, thereby eliminating blind spots in traditional scheduling; (2) under non-emergency flood discharge conditions, avoid prolonged operation of bottom outlet gates in the 2.0–3.0 m range, adopting a “rapid transit through sensitive zone” strategy, preferentially operating in the high-efficiency small opening zone or fully open state thereby reducing flow pulsation impacts on downstream energy dissipation structures.

### Energy dissipation facility optimization

#### Problems with original stilling basin design

The stilling basin is a critical energy dissipation structure, the design of which directly affects flood discharge safety and downstream channel scour protection^24^. Model tests simulated the energy dissipation performance of the combined surface-bottom outlet stilling basin for the original EG Reservoir design. The tests revealed the following deficiencies in the original layout: (1) flow entering the stilling basin under all operating conditions forms swept-out hydraulic jumps with poor energy dissipation; (2) stilling basin sidewall heights are insufficient, with water surfaces occasionally overtopping the walls; (3) under check conditions, water surface fluctuations at the stilling basin end and downstream tailwater channel are severe, with asymmetric main flow causing relatively serious scour of the downstream channel.

#### Optimized design scheme

Multiple alternative schemes were evaluated to address the deficiencies of the original stilling basin: (a) adding auxiliary energy dissipators (baffled blocks and chute blocks) within the existing stilling basin; (b) extending the stilling basin length while maintaining the original depth; and (c) the adopted “deepened pool floor + raised end sill” scheme. Comparative analysis revealed that adding auxiliary dissipators improved energy dissipation moderately but introduced risks of cavitation damage under high-velocity flow conditions and increased maintenance requirements. Extending the basin length was constrained by downstream topography and would require significantly greater excavation volumes without proportionally improving hydraulic jump stability. In contrast, the “deepened pool floor + raised end sill” scheme most effectively addressed the root cause of instability (insufficient tailwater depth for jump formation) by directly increasing the effective pool depth to ensure submerged jump conditions across all operating scenarios. Preliminary model tests confirmed that this scheme achieved the optimal balance of energy dissipation performance, structural feasibility, and construction economy.

**Fig. 7:**
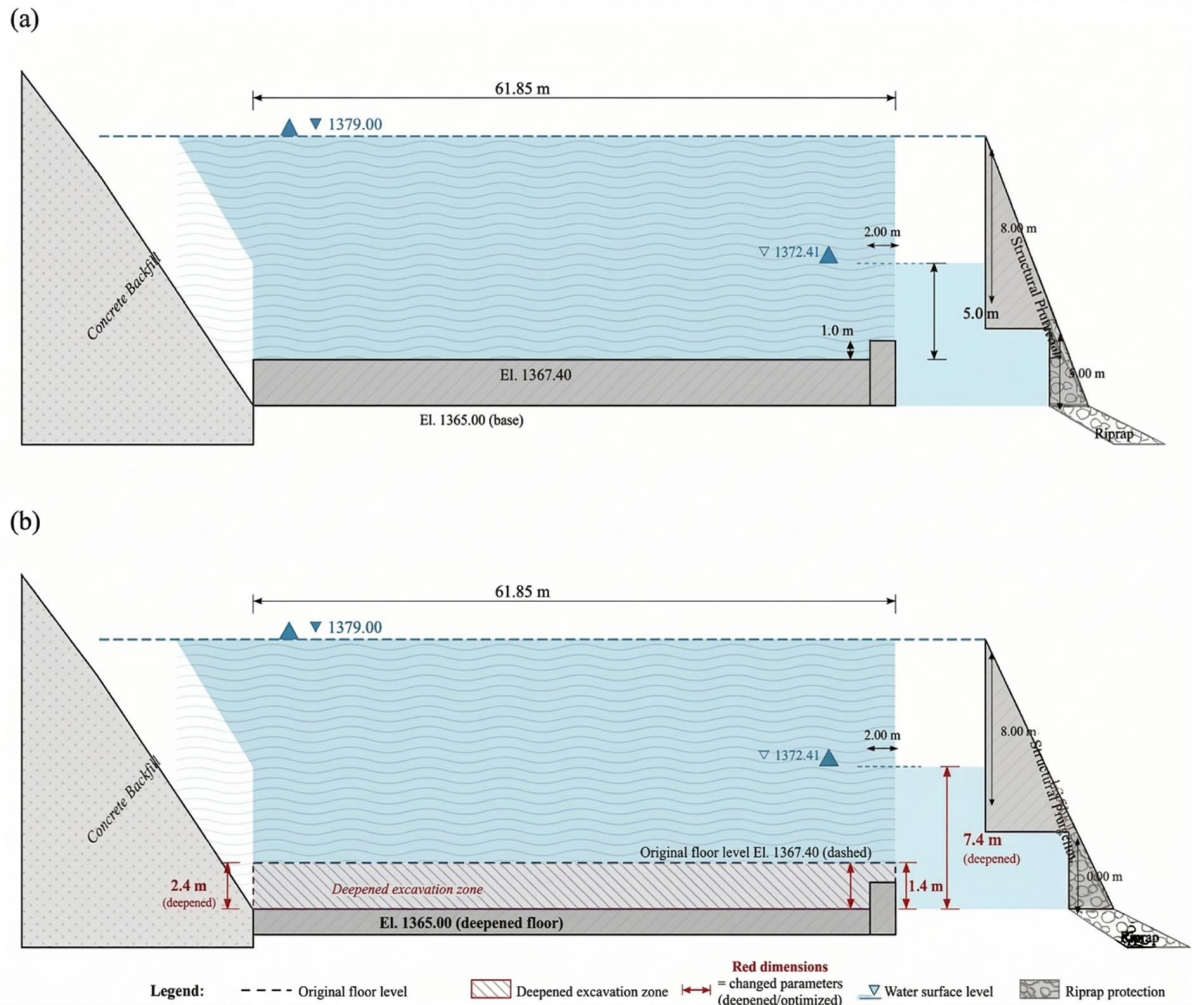
Cross-sectional comparison of the bottom outlet stilling basin before and after optimization. (**a**) Original design with pool floor at El. 1367.40 m and 1.0 m end sill, where flow formed unstable swept-out jumps. (**b**) Optimized “deepened pool floor + raised end sill” design with pool floor deepened to El. 1365.00 m and end sill raised to 1.4 m, achieving stable submerged jumps. Hatched zone indicates the deepened excavation relative to the original floor level (dashed red line). Red dimensions highlight the modified geometric parameters.

Accordingly, a combined optimization scheme of “deepened pool floor + raised end sill” was proposed: the pool floor was further excavated by 2.4 m below the original stilling basin level, increasing total pool depth from 5.0 m to 7.4 m; simultaneously, the end sill height was raised from 1.0 m to 1.4 m (Fig. 7).

Hydraulic mechanism of optimization measures: The 2.4 m floor deepening directly increases effective stilling basin depth, transforming the hydraulic jump from the unstable “swept-out jump” of the original scheme to a stable “submerged jump”. Submerged hydraulic jumps dissipate kinetic energy through intense shear turbulence between surface return flow rollers and bottom main flow^25^. Raising the end sill to 1.4 m further enhances tailwater constraint within the pool, intensifying the roller action at the sill and increasing energy losses. Additionally, the deepened pool floor–raised end sill combination effectively prevents jump front migration out of the pool under high flows such as check floods; moreover, due to the overall increased water depth, even when the bottom outlet operates in the “sensitive opening zone” causing inflow condition fluctuations, the optimized stilling basin provides greater energy dissipation safety margins, ensuring that hydraulic jumps form and develop stably within the pool.

#### Energy dissipation rate calculation and comparison

A key performance indicator for energy dissipation facilities is the energy dissipation rate^23^, calculated as:

7 $$\:\mathrm{k}\mathrm{=}\frac{{\mathrm{E}}_{\mathrm{1-}}{\mathrm{E}}_{\mathrm{2}}}{{\mathrm{E}}_{\mathrm{1}}}\mathrm{=1-}\frac{{\mathrm{E}}_{\mathrm{2}}}{{\mathrm{E}}_{\mathrm{1}}}$$

where *E*₁ is total energy at the pre-jump section and *E*₂ is total energy at the post-jump section. The total energy at the pre-jump section is calculated as *E*₁=*z*₁+*h*₁+*v*₁²/(2*g*), where z₁ is the elevation of the basin floor at the jump toe, h₁ is the conjugate (pre-jump) depth, and v₁ is the mean velocity at this section determined from continuity (*Q/A*₁). Similarly, the total energy at the post-jump section is *E*₂=*z*₂+*h*₂+*v*₂²/(2*g*), evaluated at the end sill location where z₂ is the floor elevation, h₂ is the post-jump (sequent) depth, and v₂ is the corresponding mean velocity. All water depths and velocities were measured directly in the model and converted to prototype values using the scale relationships. The energy dissipation rates of the stilling basin before and after optimization are presented in Table 4.

**Table 4:** Energy dissipation rates of the stilling basin before and after optimization.

Stilling basin configuration	Operating condition	Energy dissipation rate of surface outlet(%)	Energy dissipation rate of bottom outlet(%)
Original design (basin depth 5 m, sill height 1 m)	Design flood	51.3	46.3
	Check flood	54.1	49.2
Optimized design (basin depth 7.4 m, sill height 1.4 m)	Design flood	58.7	64.5
	Check flood	63.3	59.8

The optimized scheme significantly improved stilling basin energy dissipation performance under both operating conditions. Under design flood conditions, the bottom outlet energy dissipation rate increased from 46.3% in the original scheme to 64.5%, an increase of 18.2% points; under check flood conditions, the bottom outlet energy dissipation rate increased from 49.2% to 59.8%, an increase of approximately 10.6% points. Surface outlet energy dissipation rates also improved. This demonstrates that the deepened pool floor + raised end sill design more effectively dissipates flood discharge energy, substantially reducing the residual kinetic energy of flow exiting the pool.

The 18.2%-point improvement in energy dissipation under design flood conditions translates to a substantial reduction in residual kinetic energy entering the downstream channel. Model observations confirmed that the optimized scheme reduced the maximum downstream scour depth by approximately 35–40% compared to the original design, with the scour hole migrating closer to the end sill and exhibiting a more symmetrical profile, indicating improved flow uniformity.

Further comparison indicates the optimized stilling basin achieves energy dissipation rates exceeding 60% under design flood conditions and maintains rates above 59% under check flood conditions, approaching the upper limit of energy dissipation levels for similar small reservoir stilling basins in China (typically 50%–65%)^24^. This confirms that the proposed optimization elevates the EG Reservoir stilling basin energy dissipation performance to a high standard, demonstrating the effectiveness of the design measures.

## Discussion

### Implications of nonlinear discharge characteristics for reservoir safety

The systematic deviation between measured discharge coefficients and the constant values recommended by design codes highlights a critical gap in traditional flood routing calculations. Although standard practice assumes a constant coefficient (typically *µ =* 0.85) for safety margins, this study demonstrates that such simplifications are inadequate during partial gate operations. Specifically, the “*U*-shaped” variation pattern reveals that during intermediate openings (typically *G* = 2.0–3.0 m), the actual discharge capacity drops approximately 20% below design expectations due to intensified vertical contraction.

The implications of this “sensitive zone” are two-fold. First, regarding flood routing, reliance on static coefficients may lead to dangerous overestimations of release capacity, potentially compromising reservoir stage control during emergency scheduling. Specifically, a 21% overestimation of discharge capacity (when using a constant *µ* = 0.85 instead of the actual *µ* ≈ 0.67 in the sensitive zone) would result in a corresponding underestimation of the peak reservoir water level during flood routing. For the EG Reservoir, this deviation translates to a potential water level underestimation on the order of 0.3–0.5 m, which is significant for a small reservoir with limited freeboard. The polynomial correction model proposed herein (R^2^ *>* 0.99) provides a practical tool for correcting these deviations in real-time SCADA systems. Second, the identification of this zone suggests a refined operational strategy: operators should aim to traverse the 2.0–3.0 m range rapidly, favoring small openings for precise regulation or full openings for maximum discharge to maintain hydraulic efficiency.

### Comparison with existing literature

The *U*-shaped discharge coefficient variation identified herein can be contextualized against prior research findings. Recent computational studies^13–15^ on various gates including radial gates have shown that the curved lip geometry produces more complex contraction patterns than planar gates, consistent with our experimental observations of intensified vertical contraction in the *G* = 2.0–3.0 m range. Recent investigations on reservoir bottom outlets^16,17^ have documented nonlinear coefficient behaviors qualitatively similar to our findings, though without systematic quantification of the sensitive zone boundaries or development of polynomial correction models. The present study extends existing knowledge by: (i) providing the first quantified characterization of the *U*-shaped evolution with defined sensitive zone boundaries for a radial-gated bottom outlet; (ii) deriving a high-precision correction model directly applicable to engineering practice; and (iii) demonstrating the coupling between discharge coefficient nonlinearity and downstream energy dissipation optimization.

### The coupling of discharge fluctuations and energy dissipation

A key finding of this study is the intrinsic coupling between upstream gate hydrodynamics and downstream energy dissipation stability. Previous studies often treat the stilling basin as a passive receiver of fixed inflow; however, the present results indicate that nonlinear discharge behavior in the “sensitive zone” exacerbates hydraulic instability downstream. The rapid variation of *µ* in the sensitive zone (a coefficient range of approximately 0.64–0.70 versus 0.77–0.82 outside this zone) leads to inflow discharge variations of approximately 15–20% for the same head conditions, which directly affects the conjugate depth ratio and hydraulic jump stability within the stilling basin. This mechanism likely contributed to the unstable swept-out hydraulic jumps observed in the original stilling basin design.

Accordingly, the stilling basin optimization was not merely a geometric exercise but a necessary response to these variable inflow conditions. The “deepened pool + raised end sill” configuration successfully countered the kinetic energy spikes associated with the sensitive zone. By transforming the flow regime from a swept-out jump to a stable submerged jump, the optimized design increased the energy dissipation rate to nearly 65%. This performance aligns with the upper efficiency limits of similar hydraulic projects in China, confirming that increasing the pool depth provides the necessary hydraulic inertia to buffer the fluctuations caused by the radial gate’s nonlinear discharge.

### Limitations and future directions

Although the correction model provides high precision for the EG Reservoir, several limitations warrant acknowledgment. First, the correction model is calibrated specifically for the EG Reservoir’s deep short-tube bottom outlet with radial gate control, and its transferability to different gate types (e.g., planar gates), conduit geometries, or larger-scale projects requires independent validation before broader application, as differing boundary conditions will alter the flow contraction mechanisms. Second, all tests were conducted under clear-water conditions; thus, the influence of sediment-laden flow on the discharge coefficient and energy dissipation performance remains unquantified, which is a crucial consideration since sediment concentration can significantly modify fluid viscosity and turbulent energy dissipation dynamics. Third, the model study assumes steady-state conditions for each gate opening, whereas real-world flood routing involves transient gate operations whose dynamic effects may further modify the discharge characteristics. Fourth, despite satisfying the Froude similarity criterion, residual scale effects on turbulence intensity and air entrainment within the stilling basin cannot be entirely excluded at the 1:45 scale, potentially leading to minor discrepancies between the physical model predictions and the actual prototype performance.

Future research should pursue the following directions: (a) three-dimensional numerical simulations using Reynolds-Averaged Navier–Stokes (RANS) models with the RNG k-ε turbulence closure for initial parametric studies, with Large Eddy Simulation (LES) employed for detailed analysis of the gate lip region and the hydraulic jump roller zone to elucidate the fine-scale vortex structures and pressure fluctuations within the hydraulic sensitive zone; (b) sediment–water coupling experiments to assess the impact of sediment concentration and grain size on both the discharge coefficient and stilling basin scouring behavior; (c) multi-gate combined operation tests to evaluate the interaction effects when surface and bottom outlets operate simultaneously at various partial openings and their collective influence on energy dissipation performance; and (d) prototype field measurements during actual flood events to validate the correction model under real operating conditions.

## Conclusions

This study investigated the integrated hydraulics of the EG Reservoir flood discharge system using a 1:45 scale physical model. The principal findings are as follows:

(1) *U*-shaped nonlinear discharge coefficient variation: The discharge coefficient (*µ*) of the radial-gated bottom outlet follows a distinct *U*-shaped trajectory with gate opening (*G*). High discharge efficiency was maintained at small openings (*G* < 2.0 m, *µ* = 0.77–0.82) and partially recovered at large openings (*G* > 3.0 m, *µ* = 0.69–0.76). A hydraulic sensitive zone was identified at intermediate openings (*G* = 2.0–3.0 m) where intensified vertical contraction by the radial gate lip drives *µ* to a minimum of 0.67, approximately 21% below the constant code-recommended value of 0.85. This deviation may cause water level underestimation of 0.3–0.5 m during flood routing for the EG Reservoir, posing significant risk for a small reservoir with limited freeboard.

(2) Polynomial correction model: A cubic polynomial correction model was derived for gate openings *G* = 0.96–4.80 m. This model substantially mitigates the systematic ~ 21% calculation deviation associated with constant coefficients in the sensitive zone and is suitable for embedding into dam scheduling SCADA systems for real-time discharge correction for similarly configured structures.

(3) Operational strategies: Prolonged regulation in the sensitive zone should be avoided (*G* = 2.0–3.0 m) to minimize flow instability. A “rapid transit through sensitive zone” strategy is recommended, prioritizing high-efficiency small openings for precise regulation or full openings for maximum discharge. This strategy reduces inflow discharge variations from 15 to 20% to less than 5%, substantially improving downstream hydraulic jump stability.

(4) Stilling basin geometric optimization: A “2.4 m deepened pool floor + 0.4 m raised end sill” scheme transformed the flow regime from an unstable swept-out jump to a stable submerged jump. The energy dissipation rate under design flood conditions increased from 46.3% to 64.5% (an 18.2%-point improvement), and the maximum downstream scour depth was reduced by 35–40% with improved scour symmetry. This performance approaches the upper efficiency limit (50–65%) for similar small reservoir stilling basins in China.

These findings demonstrate that flood discharge system safety depends on treating the gate discharge coefficient and stilling basin design as coupled rather than isolated variables. The proposed correction model and geometric optimizations establish a quantitative framework for improving the engineering design of similar small-to-medium reservoirs equipped with radial-gated bottom outlets.

## Data Availability

Data will be made available on request. The datasets generated or analyzed during the current study are available from the corresponding author, Dr. Yongshuai Yan (Email: yanyongshuai@ncwu.edu.cn), on reasonable request.
